# An analysis of contractile and protrusive cell behaviors at the superficial surface of the zebrafish neural plate

**DOI:** 10.1002/dvdy.70001

**Published:** 2025-02-22

**Authors:** Claudio Araya, Raegan Boekemeyer, Francesca Farlie, Lauren Moon, Freshta Darwish, Chris Rookyard, Leanne Allison, Gema Vizcay‐Barrena, Roland Fleck, Millaray Aranda, Masa Tada, Jonathan D. W. Clarke

**Affiliations:** ^1^ Laboratory of Developmental Biology, Instituto de Ciencias Marinas y Limnológicas, Facultad de Ciencias Universidad Austral de Chile Valdivia Chile; ^2^ Centre for Developmental Neurobiology King's College London London UK; ^3^ Centre for Ultrastructural Imaging King's College London London UK; ^4^ Department of Cell and Developmental Biology University College London London UK

**Keywords:** neural plate, neurulation, zebrafish

## Abstract

**Background:**

The forces underlying convergence and internalization of the teleost neural plate remain unknown. To help understand this morphogenesis, we analyzed collective and individual cell behaviors at the superficial surface of the neural plate as internalization begins to form the neural keel in the hindbrain region of the zebrafish embryo.

**Results:**

Convergence to the midline is not accompanied by anteroposterior elongation at this stage, and it is characterized by oscillatory contractile behaviors at the superficial surface of the neural plate, a punctate distribution of Cdh2 and medially polarized actin‐rich protrusions at the surface of the neural plate. We also characterize the intimate relationship and dynamic protrusive cell behaviors between the surfaces of the motile neural plate and the stationary overlying non‐neural enveloping layer.

**Conclusions:**

Superficial neural plate cells are coupled by a punctate distribution of Cdh2‐rich adhesions. At this surface, cells tug on neighbors using oscillatory contractions. Oscillatory contractions accompany convergence and shrinkage of the cells' superficial surface for internalization during keeling. Some shrinkage for internalization occurs without oscillations. The deep surface of the overlying non‐neural enveloping layer is in contact with the superficial surface of the neural plate, suggesting that it may constrain the neural plate movements of convergence and internalization.

## Key Findings

- We characterise the punctate distribution of Cdh2 at the surface of the zebrafish neural plate.
- We characterise the oscillatory contractions of cells at the surface of the zebrafish neural plate in wt and Cdh2 mutants.
- We use high temporal and spatial resolution timelapse microscopy and serial block face scanning electron microscopy to analyse the intimate relationship and dynamic interface between the motile neural plate and overlying stationary EV

## 1 INTRODUCTION

The early stages of neural tube formation are characterized by collective cell activities affecting neural plate narrowing and internalization beneath the protective non‐neural ectoderm. In past years, accumulating evidence shows that convergent and extension movements and apical constriction are the two dominant cell behaviors responsible for this neural plate shaping.^1^, ^2^ Convergent and extension through mediolateral cell intercalation facilitate neural plate narrowing,^3^, ^4^, ^5^, ^6^ while apical cell constriction is a key behavior driving tissue internalization around the dorsal midline.^7^, ^8^ In addition, tensile forces generated within and around the neuroepithelium appear as key regulators for neural tube morphogenesis.^9^, ^10^, ^11^ The precise cellular and subcellular details of these neural plate cell dynamics remain poorly understood.

In recent years, live imaging studies in invertebrate systems have revealed that collective cell deformation is not driven by smooth continuous cell shape changes but through oscillatory steps of cell contractions.^12^, ^13^, ^14^, ^15^, ^16^, ^17^, ^18^ Key to this oscillatory cell behavior is a dynamic actomyosin network, which confers pulsatile tensile forces during cell and tissue remodeling.^15^, ^19^, ^20^, ^21^, ^22^, ^23^, ^24^ Furthermore, junctional cell–cell adhesion components like classical cadherins appear as critical components integrating actomyosin contractile forces at both cell and tissue levels.^25^ Thus, coupled pulsatile contractions have emerged as a key driver for invertebrate tissue remodeling, and although less deeply understood during vertebrate morphogenesis, oscillatory cell contractility appears to be a conserved feature in vertebrate tissue remodeling.^13^, ^26^, ^27^, ^28^

In this study, we aim to improve our understanding of vertebrate tissue narrowing and internalization by characterizing these movements at cell and subcellular levels using in vivo imaging in the zebrafish neural plate. The zebrafish neural primordium narrows dramatically and internalizes during its transition from neural plate to the neural keel, and although the teleost neural plate has a different cytoarchitecture compared to other vertebrates^29^ it uses several morphogenetic mechanisms conserved with other vertebrates.^27^, ^30^, ^31^, ^32^, ^33^, ^34^

In common with amniote and other anamniote embryos zebrafish, neural plate convergence depends on the activity of the non‐canonical Wnt/planar cell polarity (PCP) signaling pathway.^4^, ^5^, ^6^, ^30^, ^32^, ^35^, ^36^, ^37^ The PCP pathway canonically drives convergence through mediolateral cell intercalation and anteroposterior (AP) elongation.^5^, ^38^ Intercalation and AP elongation certainly occur during early neural plate formation^39^ but whether this contributes to the convergence and internalization during neural plate‐to‐neural keel transition being uncertain. In addition, zebrafish neural plate convergence and internalization are critically dependent on the function of the cell–cell adhesion protein Cdh2.^31^, ^33^, ^40^, ^41^ At the cellular level, zebrafish neural plate cells adopt cell surface constriction strategies typical of vertebrate epithelia in order to effectively reduce their superficial surface and internalize. Myosin‐II is a critical component for superficial cell surface deformation which also depends on Cdh2 activity.^33^ Abrogation of Cdh2 results in defective myosin‐II distribution, mislocalized cell internalization events and defective neural plate morphogenesis.^33^

In this study, we used high spatial and rapid temporal in vivo imaging to study cell surface dynamics during zebrafish neural plate convergence and internalization in prospective hindbrain regions. We find that during neural plate‐to‐neural keel transition, the neural plate narrows and internalizes without AP elongation. Analysis reveals that convergence is characterized by medially orientated actin‐rich protrusions that lie on the surface of the neural plate and a punctate distribution of Cdh2 between superficial cell interfaces. Both convergence and internalization are accompanied by oscillatory contractile behaviors at superficial neural plate cell surfaces. Cdh2 is also required for the medially polarized protrusive activity of neural plate cells. Finally, we analyze the interface and cell protrusions between the neural plate and the overlying non‐neural enveloping layer (the EVL), which suggest that the EVL might play a role in neural plate morphogenesis.

## 2 RESULTS

### 2.1 Cell rearrangements during convergence to the midline

**FIGURE 1:**
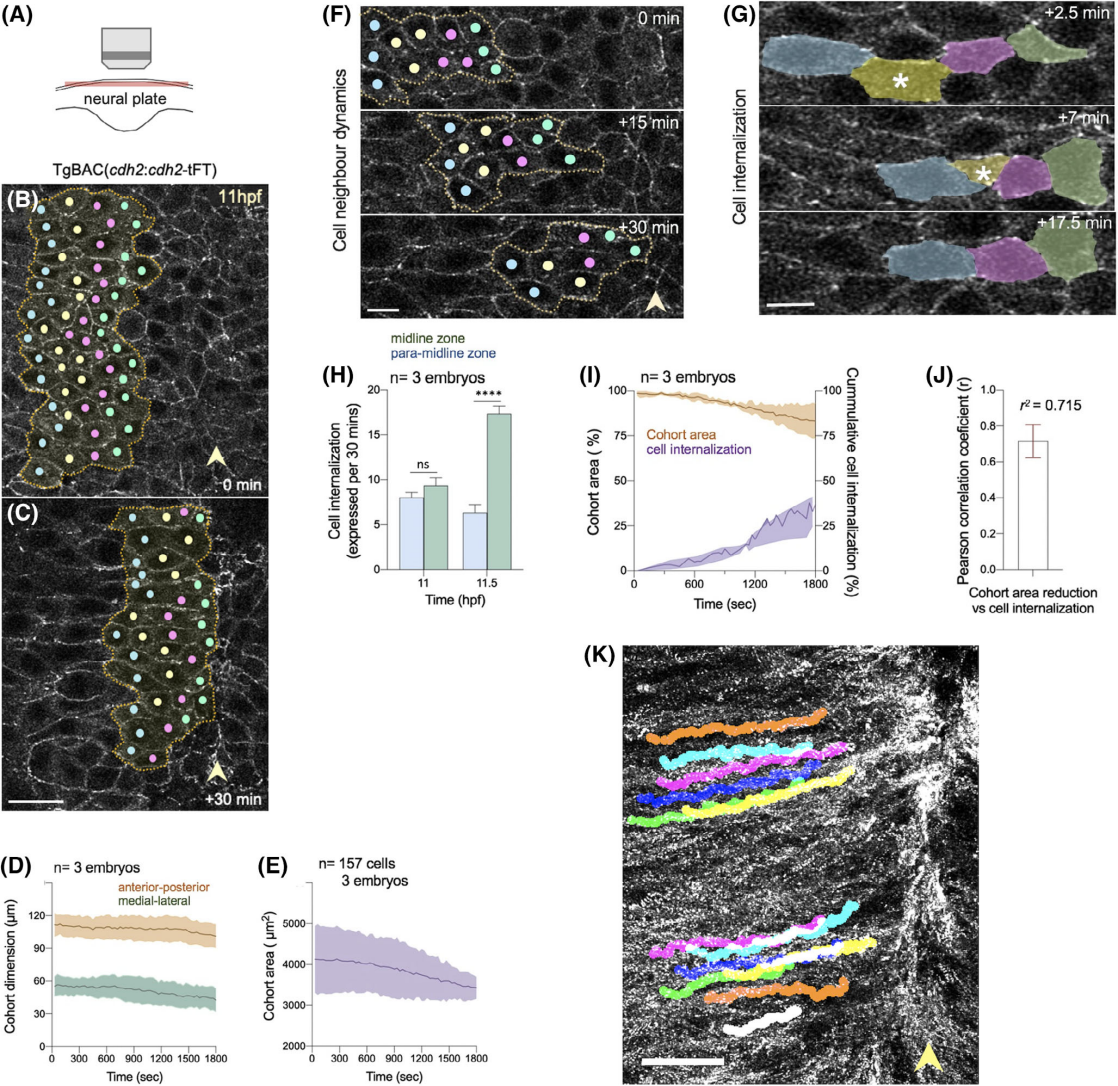
Neural plate cell dynamics during zebrafish midline convergence. (A) Schematic of imaging setup. Neural plate drawn in transverse section, but imaged from dorsal surface to give *en face* view of superficial surface. (B, C) *En face* views of superficial surface of neural plate. Two single confocal stills from a time‐lapse recording from a Cdh2‐GFP transgenic embryo between 11 and 11.5 hpf. Colored dots indicate the centroids of individual neural plate cells as they move toward the dorsal midline. Dotted lines denote the outline of a cohort of cells. Arrowheads highlight the position of neural plate midline. Up is anterior, and right is medial. Scale bar 20 μm. (D) Maximum AP and ML dimensions of cohorts during convergence (mean ± s.d., 3 embryos, average cohort *n* = 52 cells). (E) Change in area of 3 cohorts of cells during midline convergence (mean ± s.d.). (F) Example of cell neighbor relations during convergence. Scale bar 10 μm. (G) Example of cell internalization event (asterisk) during convergence. Scale bar 10 μm. (H) Cell internalization events in midline zone (pale blue) and the para‐midline zone (green) during convergence (****p <.0001 Student's *t*‐test; mean ± s.e.m., ns P = 0.4232). (I) Cohort area change (μm^2^) versus cell internalization events during midline convergence, with Pearson correlation *r* ^2^ = 0.715 ± 0.158; mean ± s.d.; *n* = 3 embryos in (J). (K) Neural plate cell tracks superimposed on background of multiple exposures of Cdh2‐GFP puncta over a time period of 30 min. Arrowhead indicates neural plate midline. Scale bar 20 μm.

To understand the movements of the neural plate during convergence and internalization, we have used time‐lapse confocal imaging to monitor the relative movements of cells at the neural plate surface in the region of the developing hindbrain between approximately 10 and 12 hpf (hours post‐fertilization). Images were acquired from the dorsal surface of the neural plate (Figure 1A) focusing on cells from the midline to approximately 70 μm more laterally. We analyzed cohorts of about 50 cells and asked whether groups of cells exhibited any of the canonical characteristics of convergent extension as they converged toward the midline. We found that cohorts of adjacent cells exhibited approximately 25% mediolateral reduction and approximately 10% anteroposterior reduction (Figure 1B–D, Movie S1). The overall area of the cohort of cells decreased by approximately 17% (Figure 1E). Cells move with a fairly constant speed of approximately 1 μm.min^−1^. Approximately 30% of cohort cells internalize in the period of our analysis (Figure 1F, Movie S1). As previously reported,^33^ cells internalize individually (Figure 1G) and most do this in the midline zone (Figure 1H) which we define as within 20 μm of the absolute midline. Because of this, the midline zone becomes characterized by a high concentration of cells with small surface profiles (Movie S1). There is a significant correlation between reduced cohort area and cell internalization (Figure 1I,J). To assess intercalation events during this period, we monitored 53 pairs of neighboring cells for approximately 1 h and found that 77% of pairs (41/53) maintained neighbor relations while 23% (12/53) became separated by an intercalating cell. Tracing the paths of individual cells as they converge to the midline shows that cells move with near parallel tracks during this period (Figure 1K, Figure S1).

### 2.2 Distribution and dynamics of Cdh2‐ and actin‐rich protrusions at the superficial NP surface

Our previous work has shown a relative accumulation of the adhesion molecule Cdh2 and the actomyosin motor complex at the superficial surface of neural plate cells. Furthermore, both Cdh2 and actomyosin activity are critical requirements for neural plate movements.^33^ To better understand their contribution to tissue and cell movements, we have used live reporters and high‐resolution in vivo imaging of their distribution and dynamics.

**FIGURE 2:**
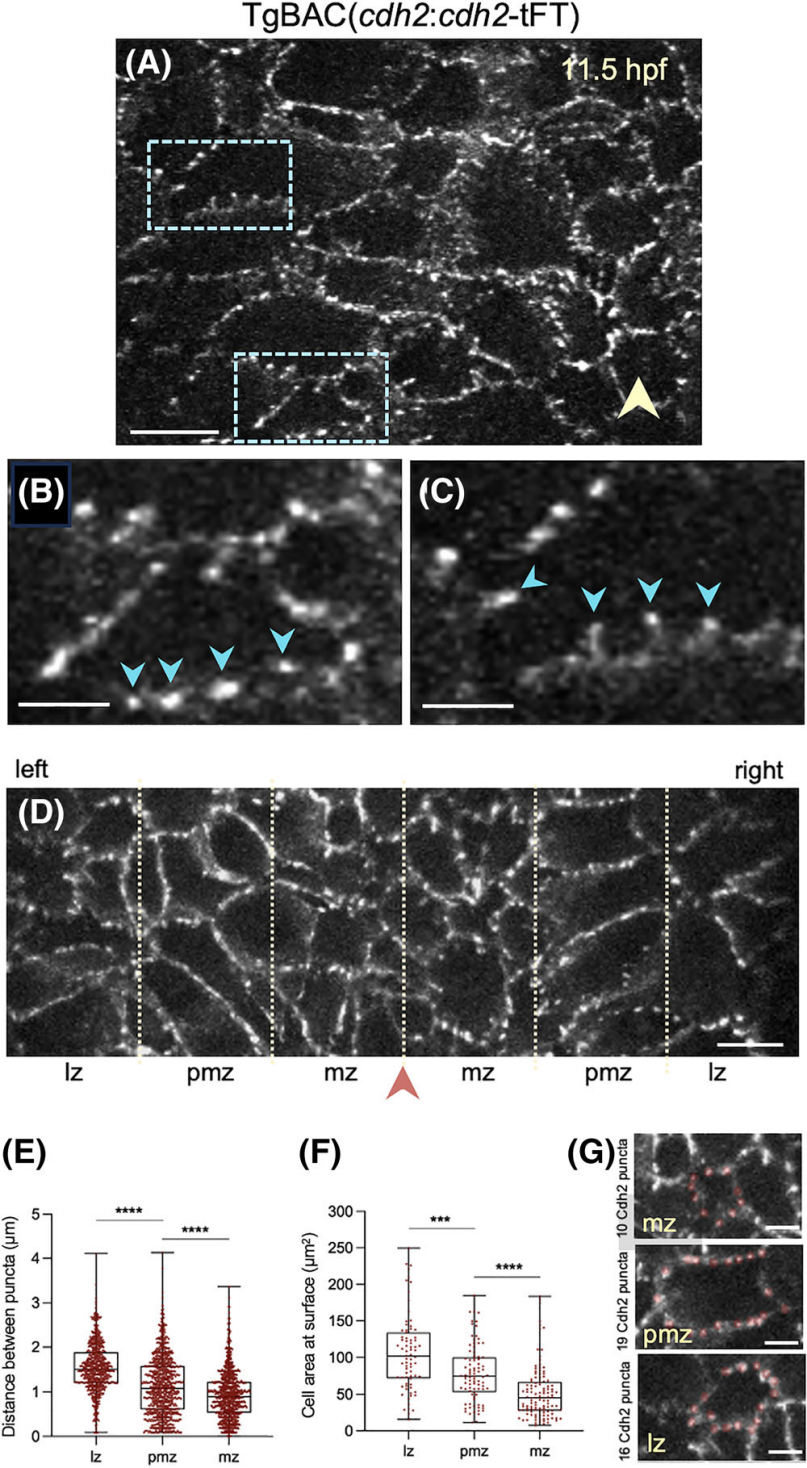
Cdh2 subcellular dynamics during midline convergence. (A) *En face* view of dorsal surface of neural plate cells (maximum projection of three z‐levels) from a Cdh2‐GFP embryo at 11.5 hpf. Yellow arrowhead indicates the midline. Anterior is up, and right is medial. Scale bar 10 μm. (B) Zoom of inset from a, depicting punctate organization of Cdh2‐GFP (blue arrowheads). Scale bar 5 μm. (C) Zoom of inset from A, depicting Cdh2‐GFP‐rich puncta on projections from cell membrane (blue arrowheads). Scale bar 5 μm. (D) Cdh2‐GFP puncta on cells within lateral zone (lz), paramedial zone (pmz), and medial zone (mz). Arrowhead indicates midline. Distance between puncta is quantified in (E), and cell area at the superficial plate surface quantifies in (F). (G) Examples of cells and puncta in the threezones. For statistics, using Mann Whitney test ****p < 0.0001 and ***p < 0.0005.

Imaging of TgBAC(*cdh2*:*cdh2*‐tFT) embryos shows that the superficial surface of neural plate cells is characterized by a punctate distribution of Cdh2 clusters. Membranes express a lower level of Cdh2 between the puncta. The puncta evenly encircle the superficial surface of individual cells, sitting at the interface with neighboring cells (Figure 2A,B). Some puncta appear to be present on small motile protrusions that sit at the periphery of the cell surfaces (Figure 2C). We quantified the distribution of Cdh2 puncta around cells and found that the distance between puncta was significantly smaller on medial cells compared to lateral cells (Figure 2D–G). By imaging thin volumes of the superficial neural plate surface with a frame interval of between 5 and 10 s, we find that the puncta are quite dynamic, appearing and disappearing, and sometimes apparently fusing with neighboring puncta (Figure S2, Movies S2 and S3). Cdh2‐GFP puncta are most prominent at the superficial surface of neural plate cells but puncta are less frequently present on deeper surfaces of the same cells where the fluorescence signal is lower and more evenly spread around the cell membranes (Figure S2).

**FIGURE 3:**
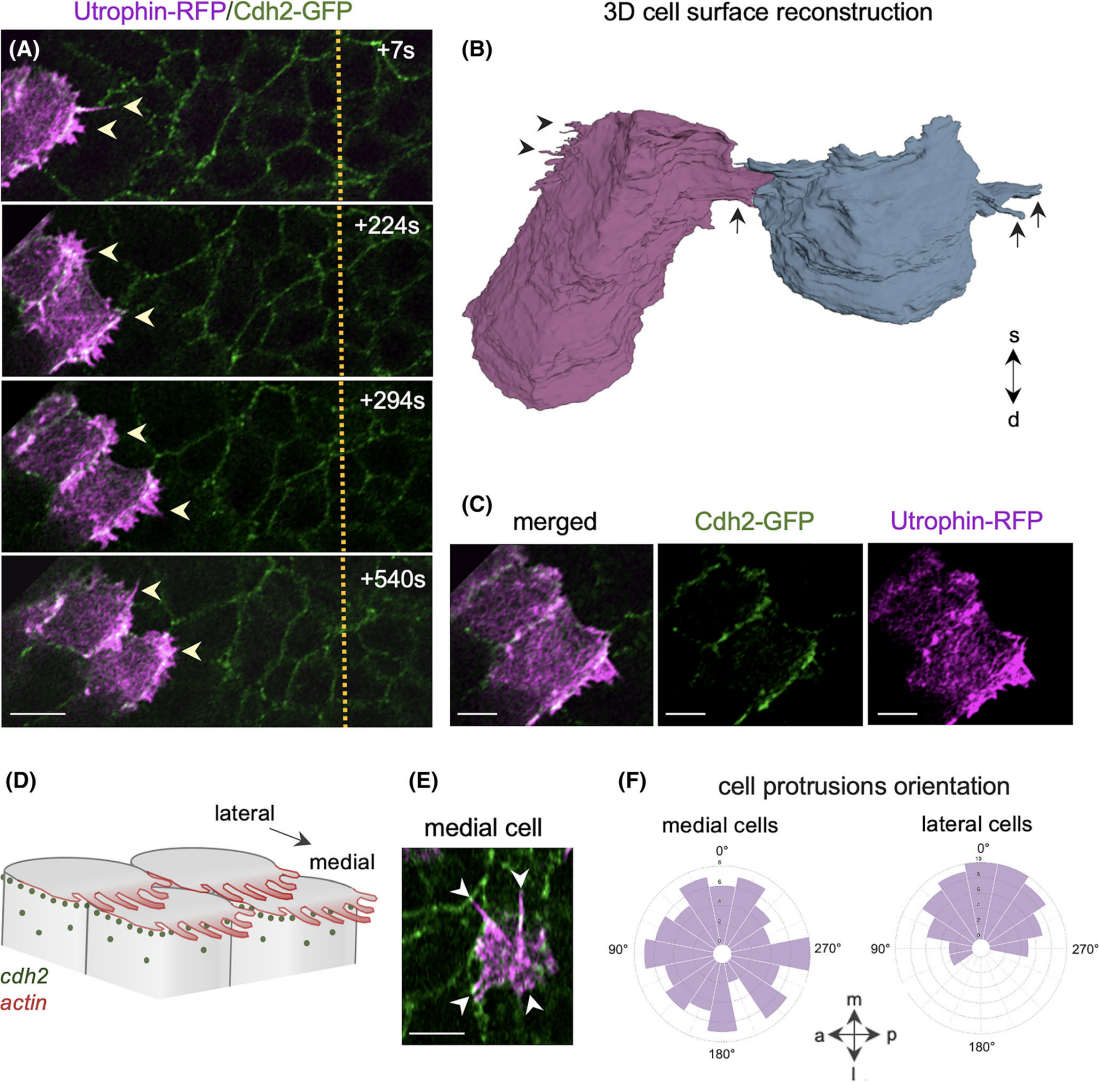
Superficial actin‐rich protrusions. (A) Four frames from time‐lapse sequence showing two neural plate cells labeled with the actin‐binding protein utr‐RFP (magenta) in a Cdh2‐GFP background at 11.5 hpf. Anterior is up, and medial is right. Dotted line denotes embryo midline, and arrowheads indicate medial‐oriented actin‐rich protrusions. Time in seconds (s), scale bar 10 μm. (B) SBFSEM 3D reconstruction of two adjacent neural plate cells during convergence. Arrows indicate midline‐oriented cell protrusions while arrowheads depict posterior oriented cell protrusions. medial to right, s‐d denotes superficial‐deep axis. (C) Separated fluorescence channels shows relative location of Cdh2‐GFP and Utr‐RFP indicating actin‐rich protrusions extend beyond Cdh2 puncta. Scale bar is 5 μm. (D) Schematic of actin‐rich protrusions and Cdh2 puncta at the superficial surface of neural plate. Only the superficial 10 μm of the cells is illustrated. (E) Cell in midline zone with more evenly distributed protrusions. Scale bar 10 μm. (F) Rose plot showing orientation of cell protrusions in medial and lateral neural plate cells (bin size, 22.5, *n* = 25 cells from 3 embryos).

By imaging the actin‐binding protein utrophin using the Tg(*actb1:GFP‐utrCH*) transgenic line, we find that the superficial surface of the neural plate is covered in extremely dynamic actin‐rich filopodia and lamellipodial protrusions. When the whole population of cells is imaged, these protrusions are seen around the superficial perimeter of every cell (Movie S4). However, when the tissue is mosaically labeled for utr‐RFP, it is clear that the actin‐rich protrusions have a very distinct polarity as they predominantly protrude medially toward the midline (Figure 3A). The protrusions lie over the surface of the neighboring (usually more medial) cell and move dynamically on this surface (Movie S5). Medially, directed protrusions are confirmed by serial block face imaging and this further shows that these protrusions lie in the plane of the interface between the neural plate and the overlying enveloping layer (EVL) (Figure 3B, see later Figure 8H,I). By imaging Cdh2‐GFP and utr‐RFP in the same cells, we find that the actin‐rich protrusions extend beyond the Cdh2 puncta (Figure 3C,D).

The polarity of actin protrusions changes when cells reach the midline zone (we define this as within 20 μm of the absolute midline). Within the midline zone, superficial protrusions are more evenly distributed around the cells' superficial perimeters (Figure 3E,F). Although we have not quantified actin‐rich protrusions at all depths of the neural plate, a qualitative assessment of fluorescence in the Tg(*actb1:GFP‐utrCH*) line suggests that they are much more prominent at the superficial surface than deeper levels of the same neural plate cells (Figure S3, Movie S6).

### 2.3 Oscillatory contractions at the neural plate surface

**FIGURE 4:**
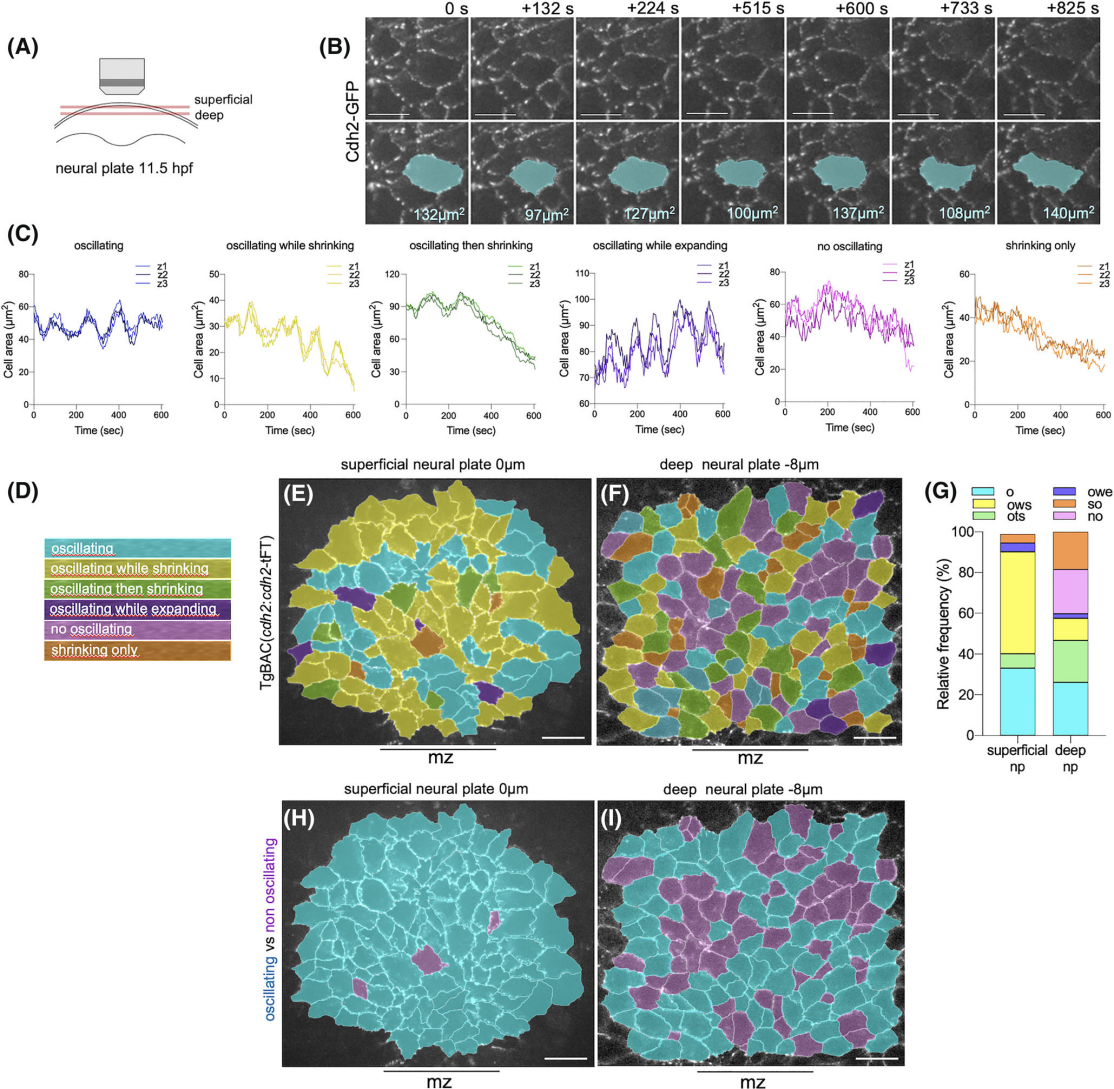
Oscillatory contractions. (A) Schematic of imaging setup. (B) Selected time‐lapse frames from superficial surface of neural plate to show dynamic cell profile. Bottom panels show changes in superficial surface area of selected cell (blue). Scale bar 10 μm. (C) Examples of contractile oscillatory behaviors in superficial cell surface area. In each graph area is measured at three consecutive z‐levels (0.5 μm apart). (D) Color code of distinctive cell surface behaviors. (E) Map of cell behaviors at superficial surface. (F) At deeper levels of the neural plate (z = 8 μm from the surface), oscillatory contractile behavior is less dominant and many cells show a non‐oscillating dynamic. In E and F, anterior is up, mz indicates midline zone. (G) Histogram of cell surface behavior at both superficial and deep levels of the neural plate. Color code indicates; o (oscillating), ows (oscillating while shrinking), ots (oscillating then shrinking), owe (oscillating while expanding), no (not oscillating), and so (shrinking only). (H, I) Maps colored to emphasize oscillating (blue) versus non‐oscillating (purple) cells. mz is midline zone and scale bar is 20 μm in (E–I).

We have visualized the detailed behaviors of individual cells at the neural plate surface by imaging the superficial cell perimeters from the dorsal surface (Figure 4A), either by imaging the Cdh2 puncta that sit at cell–cell interfaces or by imaging cell membranes directly using membrane‐GFP. Using a frame interval of between 5 and 10 s, cell perimeters at the superficial surface were seen to move in an oscillatory fashion (Figure 4B, Movie S2). Thus, cells are pulling on their neighbors in a pulsatile manner. How this behavior is coordinated across individual cells and between neighboring cells is complex and we have identified several different behaviors among and between cells.

To understand these behaviors, we analyzed the superficial surface area of cells using either Cdh2‐GFP or membrane‐GFP‐labeled cells during an approximate 10‐min period. We identified and mapped six cell behaviors (Figure 4C–E, Figure S4): cells that exhibit clear oscillations (repeated expansions and contractions of their superficial surface area) without an overall shrinkage of that cell surface; cells that oscillate and also exhibit an overall shrinkage in surface area; cells that oscillate without shrinking but then stop oscillating and shrink without oscillations; cells that oscillate but also have an overall expansion of their surface area; cells that show no clear oscillations but their surface area is noisy and may stay roughly the same size or shrink; and the predominant behaviors of cells at the superficial surface which are either oscillating (30%) or oscillating while shrinking (50%) (Figure 4H). The period of oscillations for most cells was between 2 and 3 min, and the amplitude was approximately 5%–10% of a cell's superficial surface area (Figure S3). Oscillating cells were found in all regions of the hindbrain neural plate—that is, both regions where cells converge to the midline and within the midline zone where cells internalize.

To determine whether the oscillatory behavior was particular to the superficial surface of neural plate cells, we repeated our analysis at a depth of 8 μm below the superficial surface (Movie S7). At 8 μm depth, most cell profiles will be of cells that reach the superficial surface, but a small number could be deeper cells with no attachment to the neural plate surface. In contrast to the superficial surface where only 5% of cell profiles showed no oscillatory behavior, approximately 40% of cell perimeters analyzed at 8 μm depth showed no oscillatory behavior (Figure 4F,G,I). Half of these non‐oscillating perimeters showed shrinking without oscillations. Approximately 20% of the deep cell perimeters show neither clear oscillations nor shrinking or expansion (Figure 4F,G). For this 20% of cells, their perimeters were not static but their movements were noisy, rapid, and not easily defined. For the 60% of deeper cell profiles that did oscillate, they showed similar oscillatory dynamics (frequency and amplitude) to those at the surface (Figure S4).

### 2.4 Myosin dynamics at the superficial neural plate surface

**FIGURE 5:**
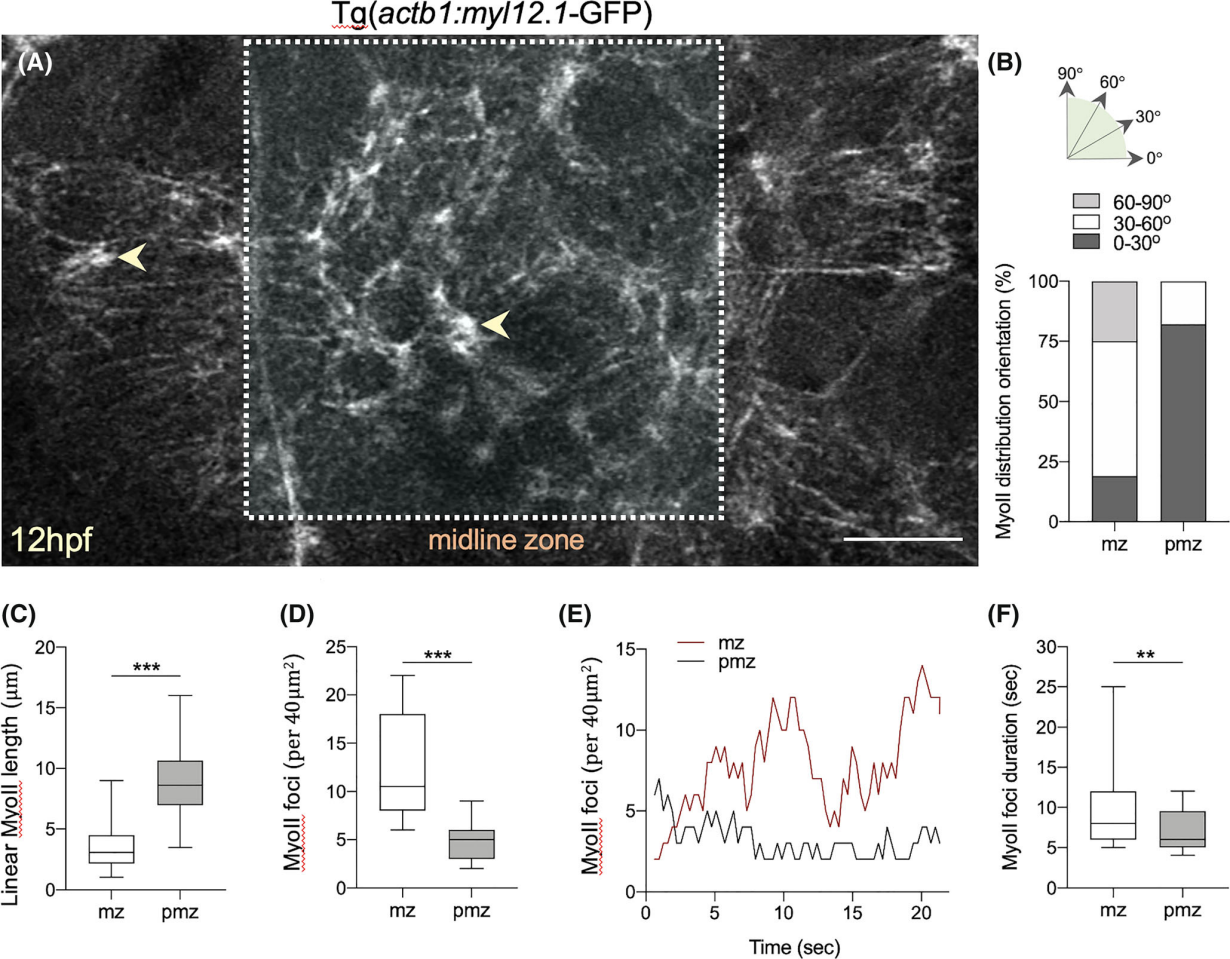
Myosin II activity at the superficial surface of the neural plate. (A) Maximal intensity confocal projection from a Tg(actb1:myl12.1‐GFP) embryo at 12 hpf. Arrowheads indicate puncta. Anterior to top, scale bar 10 μm. (B) Linear MyoII orientation in midline zone (mz) and para‐midline zone (pmz). Two embryos analyzed. (C) Length of linear MyoII in midline zone (mz) and para‐midline zone (pmz). 75 linear structures analyzed in each zone; ***p = 0.0004. (D) Number of MyoII foci (stellate organization) in midline zone (mz) and para‐midline zone (pmz). 25 foci analyzed in each zone; ***p = 0.0002. (E) MyoII foci number in midline zone (mz, red line) and para‐midline zone (pmz, black line) over 20 mins. (F) Duration of MyoII foci in midline zone (mz) and para‐midline zone (pmz). 15 foci tracked in each zone; **p = 0.009.

To help understand the contractile behaviors of cells at the superficial neural plate surface, we analyzed myosin II activity and its distribution at the subcellular level using the Tg(*actb1*:*myl12. 1*‐GFP) line. Myosin II fluorescence was very dynamic and largely appeared in three states. Cells had a uniform distribution of low‐intensity cytoplasmic fluorescence in addition to high‐intensity fluorescence organized in either a dynamic linear manner or in high‐intensity dynamic foci with a stellate appearance (Figure 5A, Movie S8). In the cells outside the midline zone, the linear myosin II was predominantly arranged in a mediolateral orientation, usually at the cell perimeters (Figure 5B). For cells within the midline zone linear, myosin II was more randomly arranged (Figure 5B). High‐intensity myosin II foci were seen throughout the superficial surface, most often at cell perimeters and often at multicell vertices. Myosin II foci with radiating linear myosin II were also seen within the center of the superficial surface of cells. Linear myosin II was longer within the para‐midline zone, and myosin II foci were more frequent in the midline zone (Figure 5C,D). The number of visible myosin II foci was more dynamic in the midline zone than in the para‐midline zone, and the duration of myosin II foci was longer in the midline zone (Figure 5E,F).

### 2.5 Neural plate dynamics and organization in cdh2^fr7/fr7^mutants

Loss of Cdh2 function results in a strong neural plate phenotype as previously reported. Convergence of the neural plate to the midline is largely inhibited, and very few midline cells internalize.^31^, ^33^, ^40^, ^41^, ^42^ But how loss of Cdh2 function results in this phenotype is not clear.

**FIGURE 6:**
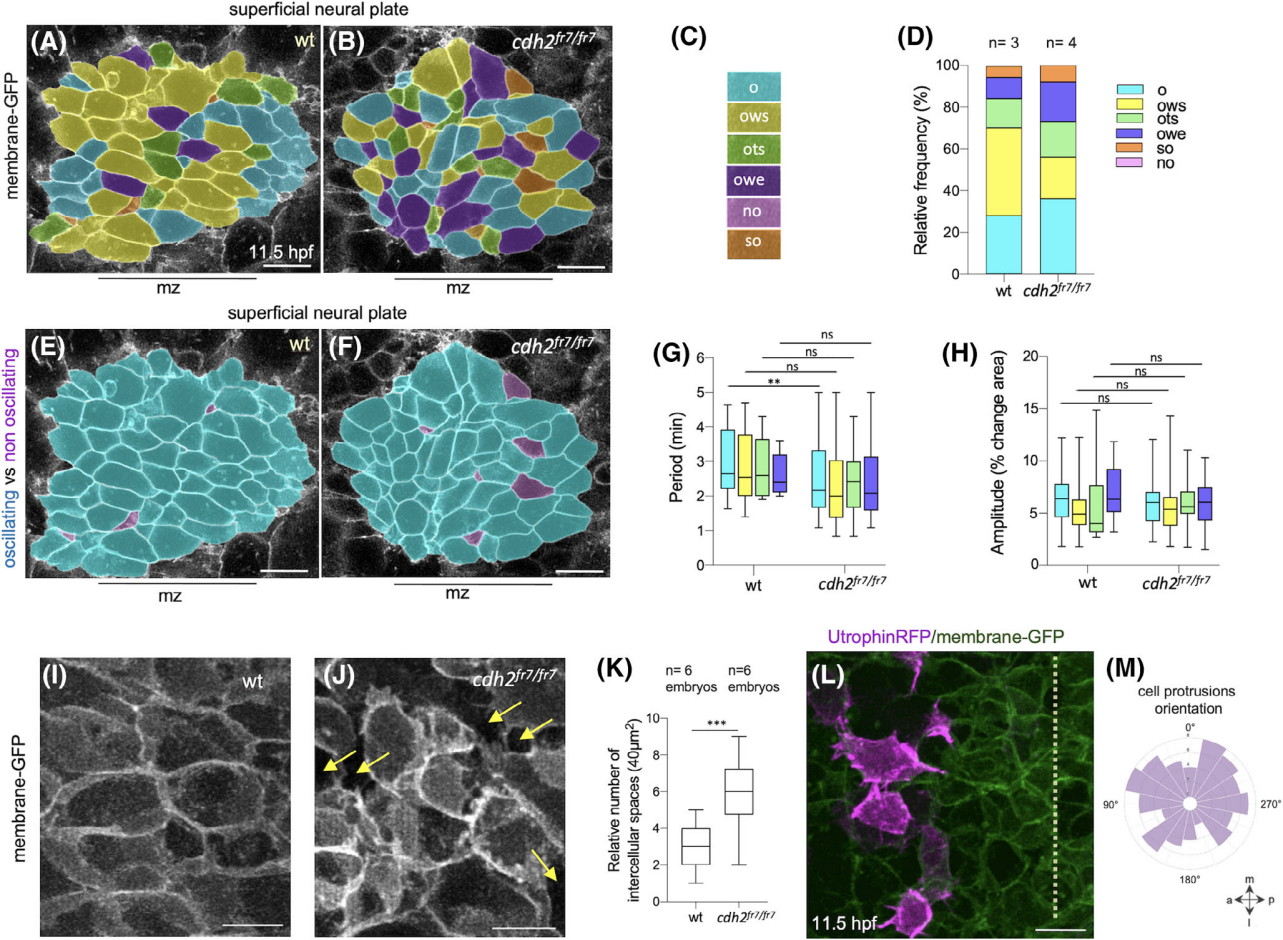
Neural plate surface dynamics in cdh2 mutants. (A, B) Maps of oscillatory activity in wt and cdh2 mutants at 11.5 hpf. Scale bar 10 μm. (C) Color code for (A) and (B); o (oscillating), ows (oscillating while shrinking), ots (oscillating then shrinking), owe (oscillating while expanding), no (not oscillating), and so (shrinking only). (D) Frequency of cell surface behaviors in wt (3 embryos) and cdh2 mutants (4 embryos). (E, F) Color code map showing distribution of oscillating (pale blue) versus non‐oscillatory (pale purple) cells in wt and cdh2 mutants. mz indicates midline zone and scale bar is 10 μm. (G) Box plot analysis comparing oscillation period between wt and cdh2 mutants. Three wt and four cdh2 embryos were analyzed, bars indicate max and min values. **p = 0.008, ns *p* > 0.05 (H) Box plot analysis comparing amplitude of oscillations in wt and cdh2 mutants. ns *p* > 0.05 (I, J) Intercellular spaces (yellow arrows) in wt and cdh2 mutants at 12 hpf. Scale bar 5 μm. (K) Box plots comparing number of intercellular spaces in wt and cdh2 mutants within a 40 μm^2^ area, ***p = 0.0002. (L) Maximum projection of utr‐RFP labeled cells (magenta) in a cdh2 mutant. Dotted line shows midline. Scale bar 10 μm. (M) Rose plot analysis depicting orientation of cell protrusions in converging cdh2 mutant cells (bin size, 22.5°, *n* = 25 cells from 3 embryos).

Neural plate cells undergo two distinct behaviors in order to transition from plate to neural keel. They must converge to the midline, and they must shrink their superficial surface area in order to internalize. During our 10‐min observation periods, 96% of all wild‐type (wt) cells oscillate and 82% of cells that shrink do so while oscillating. This could mean that oscillatory contractions are an important driver of both convergence and shrinkage. To begin to understand causality in these relationships, we quantified the oscillatory behaviors of cells in *cdh2* ^*fr7/fr7*^ mutants (Figure 6A–H, Figure S5A,B,C). We find that 92% of all neural plate cells in mutants still oscillate (Figure 6D–F) and do so with the same periodicity and amplitude as wt cells (Figure 6G,H). However, in mutants a smaller percentage of cells shrink (64% in wt compared to 45% in cdh2 mutants) and cells in the midline zone remain large compared to wt (Figure S5D,E). The percentage of cells that shrink while oscillating decreases from 40% in wt down to 20% in mutants, while the percentage of cells that shrink without oscillating is essentially the same (24% in wt and 25% in mutants). Additionally, more oscillating cells expand in mutants (10% in wt compared to 19% in mutants). These data show that when Cdh2 adhesion is defective, convergence is inhibited despite continued oscillatory contractions and that oscillating cells are less able to shrink their superficial surface if they are Cdh2 defective.

Analysis of Cdh2 mutants revealed two other changes at the superficial surface of the neural plate. One is an increase in extracellular space (gaps) between the superficial cells (Figure 6I–K, Movie S9), and the second is that the actin‐rich protrusions are less polarized toward the midline than in wt cells (Figure 6L,M, Movie S10).

Together these results show that Cdh2 adhesivity is not required for oscillatory contractions in neural plate cells but is a significant component of the mechanism that allows oscillating cells to converge and reduce their superficial surface. In addition, Cdh2 adhesivity is required for the compactness of neural plate cells and is required for the medially biased polarity of actin‐rich protrusions at the surface of the neural plate.

### 2.6 Relationship between the neural plate and the overlying EVL

**FIGURE 7:**
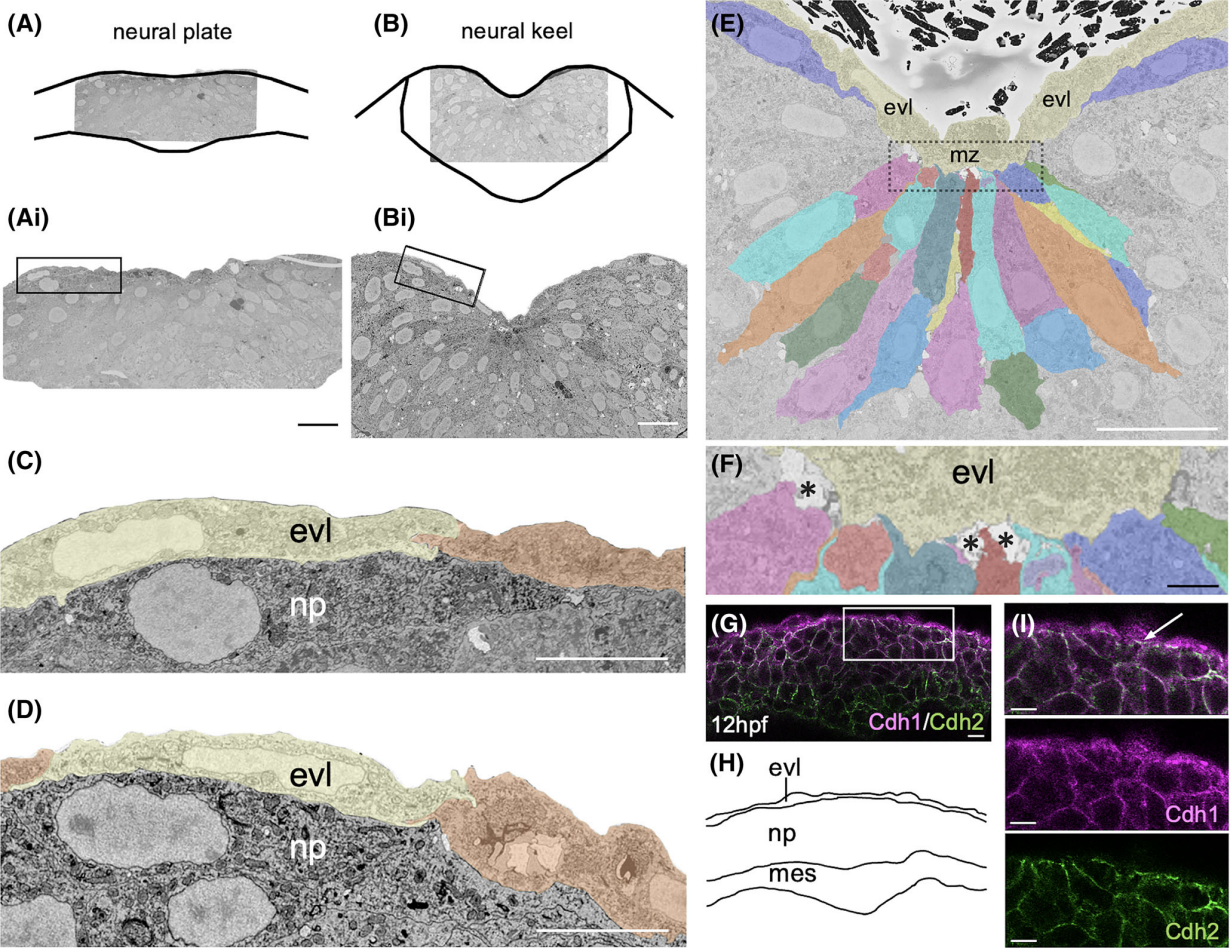
Relationship of neural plate and enveloping layer (EVL). (A, B) Schematics to show tissue orientation in serial block face image Movies S12 and S13. (Ai, Bi) Low‐mag micrographs showing regions of interest magnified in (C) and (D). Scale bar 20 μm. (C, D) Close apposition of neural plate and EVL cells at plate and early neural keel stages. Scale bar 20 μm. (E) Serial block face micrograph at 12 hpf depicting relation of neural plate cells with EVL in the midline zone (mz, box). EVL cells are tinted yellow. Scale bar 20 μm. (F) High mag of boxed region in (E). Asterisks indicate extracellular space. Scale bar 2.5 μm. (G) Transverse confocal section of the neural plate at 12 hpf stained for Cdh1 (magenta) and Cdh2 (green). Scale bar 10 μm. (H) Schematic showing tissue organization in (S). (I) High mag of inset in (G), showing relative location of Cdh1 and Cdh2 at the EVL–neural plate interface.

Light microscope images and time‐lapse movies show that the superficial surface of the neural plate is very close to and possibly in contact with the deep surface of the embryo's protective epithelium—the enveloping layer (EVL) (Movie S11). This raises the question of whether the rapidly moving neural plate adheres to the stationary EVL and what cell behaviors might allow this dynamic interaction. To examine this relationship, we used high‐resolution serial block face scanning electron microscopy (SBFSEM) imaging (Figure 7). This reveals that, in regions outside the midline zone, the deep surface of the EVL and the superficial surface of the neural plate are in intimate, near‐continuous contact with very little intervening extracellular space (Figure 7A–D, Movie S12). In contrast, at early neural keel stages, while no extracellular space is obvious between the EVL and neural plate in para‐midline locations, a distinct increase in extracellular space between the neural plate and the EVL has developed at the midline zone where cells are internalizing to form the neural keel (Figure 7E,F).

To examine the possibility that neural plate and EVL cells adhere to one another, we analyzed the distribution of the Cdh1 adhesion molecule. In contrast to Cdh2 which is not expressed in EVL, Cdh1 immunoreactivity is found at the surface of both EVL and neural plate cells and enriched at their interface (Figure 7G–I) suggesting that homophylic (Cdh1‐Cdh1) and heterophilic (Cdh1‐Cdh2) adhesions could help stick these tissues together across this interface.

**FIGURE 8:**
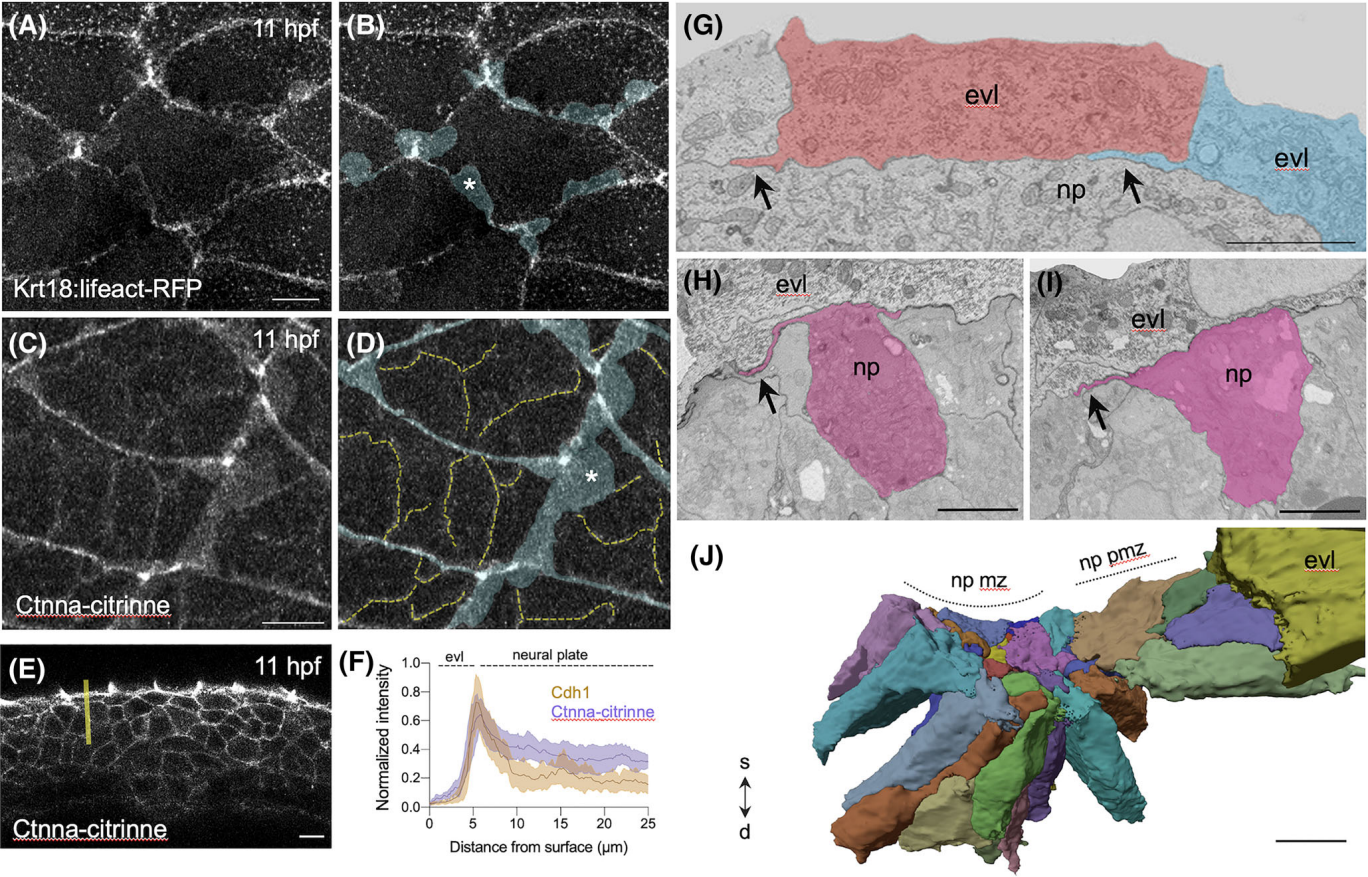
Enveloping layer (EVL) and neural plate cell projections. (A, B) Single confocal image showing basal EVL lamellar projections (pseudocolored blue in B) in Krt18:Lifeact‐RFP embryos at 11hpf. Scale bar 10 μm. (C, D) Maximum intensity projection at EVL–neural plate interface from a Tg(ctnna‐citrine)^ct3a^ embryo at 11hpf. Scale bar 10 μm. Dashed yellow lines in D denote Ctnna expression in neural plate cells and blue tint denotes Ctnna in EVL lamelli. Asterisks in B and D denote basal lamellas. (E) Transverse confocal section of the neural plate and EVL in a Tg(ctnna‐citrine)^ct3a^ embryo at 11 hpf. Yellow line indicates example of line selection for quantification of Ctnna intensity. Scale bar 10 μm. (F) Normalized intensity of Cdh1 (brown) and Ctnna (purple) signal across EVL and neural plate. (G) Serial block face image across neural plate–EVL interface in para‐medial region depicting medially‐orientated basal projections of EVL cells (arrows). Scale bar 5 μm. np is neural plate, medial to left. (H, I) Serial block face images across neural plate–EVL interface in para‐medial region depicting medially oriented protrusions from neural plate cells (tinted pink). Medial to left, scale bar 5 μm. (J) 3D reconstruction from serial block face images showing superficial surface of neural plate cells in midline (mz) and para‐midline (pm) zones. A single EVL cell is depicted in yellow, and all other EVL cells have been removed from the reconstruction. S‐D, indicates superficial‐deep axis of the tissue. Scale bar 10 μm.

The close proximity and common adhesion molecule suggest that EVL and neural plate are in intimate contact, but since neural plate cells “glide” past the stationary EVL cells (Movie S11) this interaction must be weak enough to allow rapid relative movement. To better understand how the stationary EVL cells behave in relation to the moving neural plate cells, we imaged the deep surface of the EVL to see whether it would reveal any details of how these two tissues interact. Using a reporter of actin distribution in the EVL (Tg(Krt18:lifeact‐RFP^43^)), we found that the deep (basal) surface of the EVL is rich in dynamic, actin‐rich lamellipodial protrusions that emanate from the interfaces of adjacent EVL cells (Figure 8A,B, Movie S13). These EVL protrusions and their dynamics are also clearly visible (Figure 8C,D, Movie S14) using the GT(ctnna‐citrine)^ct3a^ gene trap line.^44^ This reporter shows that Ctnna is abundant in the EVL protrusions (Figure 8E) and enriched in the neural plate/EVL interface (Figure 8E,F) suggesting a functional link between cadherins and cell cytoskeleton in these locations. The Ctnna reporter does not appear to be expressed on the superficial lamellae or filopodial protrusions from neural plate cells but is expressed in puncta at the superficial junctions between neural plate cells and more generally in the cell membranes between neural plate cells. SBFSEM observations show that the EVL protrusions lie in the plane of the interface between the EVL and neural plate (Figure 8G, Movie S12).

Earlier we showed that the superficial surface of neural plate cells is characterized by medially biased lamellipodial protrusions (Figure 3). SBFSEM imaging confirms that, like the EVL protrusions, medially directed protrusions from neural plate cells lie sandwiched along the plane of the EVL/neural plate interface (Figure 8H,I, Movie S15). Three‐dimensional reconstruction of multiple neural plate cells confirms that their superficial surface is much reduced in area and is more uneven at the midline zone compared to the larger and smoother surfaces of para‐midline zone cells (Figure 8J).

Combined with the observations of superficial protrusions from neural plate cells, these observations show that the close apposition between the neural plate and EVL cells is characterized by highly dynamic protrusions from both tissue layers. The protrusions from both layers lie sandwiched in the plane of the interface and do not protrude into the cells of the opposing layer. The distribution of Cdh1 in both EVL and neural plates suggests that these cells are likely to adhere to one another through homophylic Cdh1‐Cdh1 adhesions; additionally, heterophilic Cdh1‐Cdh2 adhesions across the tissue interface may also be possible.^45^, ^46^, ^47^, ^48^

## 3 DISCUSSION

In this work, we analyze the cell and subcellular behaviors that accompany movements of the zebrafish neural plate as it converges to the midline and internalizes to form the neural keel. Our results show that during neural plate‐to‐neural keel transition, cells move toward the midline without an accompanying AP tissue elongation. At the superficial surface, cells are mechanically coupled together through Cdh2‐rich punctate adhesions and converging cells almost continually tug on their neighbors through oscillatory surface contractions. Cells continue to oscillate in the midline zone and most internalizing cells shrink their superficial surface area while oscillating, although a small percentage shrink without oscillations. Neural plate cells in cdh2 mutants are less compact, and although they still oscillate, they are unable to converge to the midline or reduce their superficial surface to internalize. Here, we discuss the contributions that cell–cell rearrangements, oscillatory contractions, cadherin‐based adhesions and polarized cell protrusions may have during this morphogenetic process.

### 3.1 Neural plate‐to‐neural keel transition

The motive forces that drive cells in the teleost neural plate to the midline are not well understood. In common with other vertebrates, efficient narrowing of the neural plate requires signaling of the planar cell polarity (PCP) pathway^30^, ^32^ but how this controls cell and tissue movements is not certain. In other vertebrates and other tissues, PCP function during tissue narrowing is often associated with cell intercalation along the mediolateral axis in a manner that simultaneously affects mediolateral narrowing and anteroposterior elongation (thus driving convergent extension). An analysis of clonal dispersion in zebrafish shows that cells fated to be neural do separate along the AP axis by mediolateral intercalation between 5 and 10 hpf.^39^ But here we show that the subsequent narrowing of the zebrafish neural plate (during the phase of plate‐to‐keel transition in the hindbrain region) does not involve significant cell separation along the AP axis. While some cell intercalation events do occur at the plate surface, these likely do not result in AP elongation because this is counteracted by other cells internalizing to deepen the neural primordium at the midline and form the neural keel. We have only analyzed the prospective hindbrain region, but our result is reminiscent of the Xenopus anterior neural plate where mediolateral intercalation also contributes little to convergence even though it does appear significant in more posterior regions.^28^ Although cell internalization at the zebrafish midline is itself also a cell intercalation event, it does not appear to be critically regulated by the PCP pathway since although PCP depletion decreases the speed of neural plate convergence, internalization for the neural plate‐to‐neural tube transition still occurs.^32^

### 3.2 Cdh2 adhesions

In addition to PCP signaling, zebrafish neural plate morphogenesis is strongly dependent on the function of the adhesion protein Cdh2.^31^, ^33^, ^40^ Neural plate cells in Cdh2 loss of function mutants are unable to converge to the midline or internalize.^33^ Cdh2 mutant cells transplanted into a wild‐type background accumulate dorsally in the neural tube,^40^ suggesting either they cannot move medially with their wild‐type neighbors in the plate or they cannot internalize when they reach the midline zone (or both). Here we show Cdh2 protein has a particular dynamic punctate distribution around the superficial surface of all neural plate cells. We assume that these puncta are composed of Cdh2‐rich adhesions representing sites of strong homophylic adhesions between adjacent cells^49^, ^50^ and mechanically couple these cells so they can transmit the forces needed for convergence and keeling. We find that Cdh2 puncta at cell–cell interfaces are significantly closer together on medial compared to lateral cells potentially changing the tensile properties of the tissue across the mediolateral axis. There is increased extracellular space between Cdh2‐deficient neural plate cells suggesting that Cdh2 is a major factor responsible for cohesion between neural plate cells. Increased space between cells has previously been reported in other Cdh2‐deficient tissue^51^ and cadherin functions to aid cohesion during *C. elegans* neurulation.^52^ Since our current time‐lapse analysis shows that superficial NP cells almost continuously tug on each other in an oscillatory manner, we speculate a loss of adhesion between Cdh2‐deficient cells in zebrafish results in a loss of the traction and the propagating motive forces required for convergence to the midline.

### 3.3 Oscillatory behavior

Oscillatory contractions appear to be a common feature of many morphogenetic processes across many animal groups^16^, ^20^, ^22^, ^53^ including Xenopus neural plate.^54^ They have previously been briefly described in the anterior neural plate of the zebrafish,^27^ and here we quantify their dynamics and relate them to other behaviors at the surface of the zebrafish NP in the hindbrain region. The period of neural plate contractile oscillations is roughly 2.5 min and fits well with the oscillatory behaviors of other systems.^12^, ^22^ They are a more prominent feature of the superficial surface of neural plate cells than other parts of the cells deeper to the surface. They are prominent both in cells converging to the midline and in cells within the midline zone during internalization. Although oscillations are a common feature of shrinking cells, they do not appear to be a required feature of the neural plate cells' ability to shrink their superficial surface in preparation for internalization at the midline, as shrinkage occurs without oscillations in a small percentage of cells. Thus, the contractile mechanisms of oscillations and overall shrinkage can be independent of each other and able to work simultaneously or separately. Oscillations still occur with the same amplitude and frequency and in a similar proportion of cells in Cdh2 mutants, showing that Cdh2‐mediated adhesion is not required for oscillation and suggests that oscillatory contractions could be a cell autonomous feature of superficial neural plate cells.

While we currently do not understand the function of oscillatory contractions during convergence and internalization, evidence in invertebrate systems suggests that cell oscillation is associated with the formation and transmission of biomechanical properties across the tissue.^12^, ^19^ During *Drosophila* mesoderm invagination, asynchronous oscillations of apical actin–myosin are associated with the formation of supracellular actomyosin networks that confers the tensile properties across the tissue required for bending.^25^ We speculate that the oscillatory tugging between cells that adhere through cadherin‐based adhesions is required to generate the tensile strength necessary for convergence movements of the neural plate toward the dorsal midline.

### 3.4 Polarized actin‐rich protrusions

Our analyses of cell morphology using both high‐resolution time‐lapse imaging and serial block face reconstruction reveal that the superficial surface of each cell at the surface of the neural plate is morphologically polarized with dynamic lamellipodial and filopodial protrusions directed toward the midline. These protrusions are prominent at the superficial cell surface and lie in the plane of the interface between the neural plate and the overlying EVL. These protrusions appear not to express either Cdh2 or Ctnna proteins, so probably do not mediate cadherin‐based adhesion and this perhaps suggests a more sensory/exploratory role (detecting or responding to a mediolateral positional gradient?). Whatever their role, their location along the superficial surface of the neural plate suggests that this is a key location for events critical for convergence to the midline. Once cells are within the midline zone, the polarity of these actin‐rich protrusions is lost—the protrusions are still present but here they are elaborated all around the superficial surface of cells. We speculate that here they may switch their role from potentially enabling convergence to enabling internalization. Outside the midline zone, their polarity suggests that they may detect a medial to lateral positional gradient, when at the midline this gradient would disappear and the even distribution of actin‐rich protrusions could reflect their new position and trigger a new behavior (internalization). In Cdh2 mutants (where cells do not converge to the midline), the polarized distribution of non‐midline protrusions is lost (Figure 6M,N), and the protrusions are still present but no longer biased to the midline. This could suggest that Cdh2 is required for cells to know their position relative to the midline, although an alternative possibility is that loss of movement toward the midline somehow allows a redistribution of protrusions. We note that neural plate cell protrusions have been described previously in zebrafish^31^; however, the protrusive activity described in that work is likely to reflect the overall dynamic spindle shape of cells rather than the actin‐rich lamellae and filopodia we describe here at the superficial surface of the plate.

### 3.5 Cdh2 and internalization at the midline

Wild‐type cells reduce their superficial surface area in the midline zone to internalize but Cdh2‐deficient cells do not do that. A reduction in a cell's superficial surface area is presumably a prerequisite for internalization. The prominent appearance and dynamics of myosin II foci in the midline zone lead us to suggest that the reduction in surface area involves an active contractile process, but currently, we do not know whether contractions are in the internalizing cell or in its surrounding neighbors or both. Internalizing cells potentially leave a hole in the neural plate surface. This hole could be sealed simply by the shrinking cell drawing its neighbors together through cell–cell adhesions, or alternatively surrounding cells could purse‐string together to seal over the potential gap (and potentially squeezing the internalizing cell below the surface). A cooperative mechanism between surrounding cells has been proposed to seal tissue holes resulting from cell internalization during *C. elegans* gastrulation,^55^ and internalization by capping over with neighboring cells contributes to invagination in salivary glands and teeth.^56^ We find that more prominent gaps between cdh2 mutant cells and fewer mutant cells are able to shrink their superficial surface compared to wt. While 42% of cells in wt shrink while oscillating, this is reduced to only 20% of cells in cdh2 mutants. This suggests that Cdh2 adhesivity is an important component of the shrinking mechanism in oscillating cells. Additionally, since more oscillating cells in cdh2 mutants are able to expand, this potentially suggests that cells with normal Cdh2 adhesions are better able to stabilize a reduced superficial area. Perhaps, the increased extracellular space between mutant neural plate cells simply allows more cells to expand into that space or the reduced adhesion and increased gaps between cells which eliminates the cooperative mechanisms of internalization suggested above.

### 3.6 EVL/neural plate interaction

Unlike the superficial surface of the neural plate in all non‐teleost species, the superficial surface of the teleost neural plate is not a “free” surface. It is covered by a thin, squamous, non‐neural epithelium called the enveloping layer (EVL). Rather little attention has previously been given to the possible interactions between the neural plate and the overlying non‐neural EVL. Here, we show that the two tissues are in intimate contact and Cdh1 and Ctnna distributions suggest that neural plate and EVL adhere to each other. Our antibody labeling suggests that Cdh1 is expressed in membranes of both EVL and plate cells, so EVL/neural plate adhesions could be mediated by homophylic Cdh1 interactions. However, since heterophylic interactions between Cdh1 and Cdh2 have been shown in mammalian cells^46^, ^47^ and suggested in zebrafish embryos,^48^ there may also be heterophylic adhesions between the EVL and neural plate. Nonetheless, EVL/neural plate adhesions must be either relatively weak or rapidly dynamic to enable the motile plate to move against the static EVL. Our time‐lapse imaging show that this tissue interface is an extremely dynamic environment with both neural plate and EVL cells elaborating dynamic actin‐rich protrusions along the tissue interface. One possibility is that extension and retraction of these dynamic protrusions minimize the time that any individual adhesions can be retained between the two layers; that is, they potentially act as an anti‐friction mechanism between the two surfaces. The actin‐rich protrusions from EVL cells above the neural plate appear to be very similar to the basal ruffles described on marginal EVL cells at the earlier, epiboly stages of development.^57^ Basal actin‐rich protrusions from EVL cells have also been reported to aid the rearrangements of deep cells during epiboly.^58^

It seems likely that the EVL confines the movement of the converging neural plate cells—restricting the directionality of movement to the plane of the neural plate. Without the EVL, the contractile oscillations of the neural plate cells could potentially squeeze cells or parts of cells up and out of the superficial plane of the neural plate. But with the EVL lying over and in contact with the neural plate, the surfaces of plate cells are constrained to a flat surface, an arrangement potentially mechanically beneficial in moving the plate to the midline. Tensile properties of non‐neural ectoderm have previously been suggested to aid the movements of neurulation in Xenopus embryos,^28^, ^59^ but in that case the non‐neural ectoderm lies alongside the neural plate in contrast to the EVL which sits directly above the zebrafish neural plate. Finally, when cells reach the midline zone, they internalize, so any adhesions between the EVL and neural plate within this zone must be lost and this is potentially reflected in the increase in extracellular space that we observe between plate and EVL at the midline (Figure 7E,F).

## 4 EXPERIMENTAL PROCEDURES

### 4.1 Animals

Adult zebrafish (*Danio rerio*) were kept at standard conditions at 28.5°C water with a 14‐h/10‐h photoperiod light cycle^60^ at both the King's College London (KCL) and Universidad Austral de Chile (UACh) Fish Facilities. Wild‐type AB, TgBAC(*cdh2:Cdh2*‐tFT),^61^ *cdh2* ^*fr7/fr7*^ */pac*,^40^ Tg(*actb1:myl12*.*1*‐GFP),^62^ Tg(*actb1:GFP‐utrCH*),^63^ Tg(*ctnna‐citrine*)^*ct3a*^,^44^ and Tg(Krt18:lifeact‐RFP)^43^ zebrafish lines were used in this study. Embryos were raised in E3 medium and staged by morphology according to Kimmel et al.^64^ Developmental stages were given in terms of hours post‐fertilization (hpf). All animal experiments were performed in compliance with the guidelines and approved by animal ethics committees of both KCL (Home Office Animals, Scientific Procedures, Act 1986, ASPA) and by UACh/DID animal committee.

### 4.2 mRNA synthesis and microinjection

pCS2+ expression vectors carrying membrane‐localized fluorescent CAAX‐GFP (mGFP) or utrophin‐RFP (utr‐RFP) were linearized using restriction enzymes (Promega) for 2 h at 37°C and precipitated at −20°C overnight in 70% ethanol. mRNA in vitrotranscription was performed using the SP6 message machine kit (Ambion, AM1340), purified through a column (Roche), and final concentrations were measured using a Nanodrop 3300 spectrometer (Thermoscientific). Embryo microinjections were performed under a dissecting microscope using a glass slide and petri dish.^60^ Microinjections were performed using a glass micropipette with filament (Harvard Apparatus) coupled to a micromanipulator and attached to a Picospritzer® (General Valve Corporation). mRNA was injected at 80–100 pg. per embryo. For ubiquitous localization, mRNA was injected at one‐cell stage. For mosaic injection, mRNA was injected into a single blastomere at 128‐cell stage embryo.

### 4.3 Live confocal imaging

Embryos at neural plate stages were mounted in 1.5% low‐melting‐point agarose in a glass‐bottomed imaging chamber and filled with embryo medium at 28.5°C and supplemented with tricaine at 0.05% (Sigma). Embryos were orientated dorsal uppermost at the level of the hindbrain. Images acquired on a Zeiss LSM 880 Fast Airyscan microscope provided with heated stage at 28.5°C and imaging with water dipping × 20/1.0 NA objective lens. Time‐lapse images were most frequently taken with a frame interval of 5–10 s and z‐stack depth of approximately 5–10 μm and 0.3 μm z‐resolution. All raw Airyscan images were processed with Zeiss Airyscan processing software.

### 4.4 Cell rearrangements during convergence

Cohorts of approximately 50 cells arranged in roughly four rows were followed during convergence to the midline. Changes to cohort dimensions were analyzed by measuring the maximum anteroposterior and maximum mediolateral dimensions at each time point. Total cohort area was measured by outlining the irregular edges of cohorts of cells at each time point.

### 4.5 Imaging processing and oscillation analysis

Imaging processing was carried out using ImageJ software. To track cell movements over time, we used the Manual Tracking plug‐in using the cell centroid as reference. Projections of z‐stacks are maximum projections unless otherwise indicated. To measure the duration of time individual, Cdh2 puncta were expressed at the cell membrane, and movies with 5‐s time intervals were used. Cdh2 puncta (*n* = 105) were randomly selected and tracked frame by frame, from the frame of first appearance to the frame of the Cdh2 puncta that were last visible in. The absolute value for the number of Cdh2 puncta present at the cell boundary for each frame was counted. To quantify the number of Cdh2 puncta with respect to the length of the membrane, the cell perimeter was measured for the same cells previously used to count the number of Cdh2 puncta. The cell perimeter was then divided by the number of Cdh2 puncta present in that frame, calculated in microns per puncta, and an average across all time points was made for each cell. Pixel intensity quantification for Figure 8C was carried out as previously described in detail.^33^ To extract cell plasma membrane dynamics over time, we used freehand tool to manually segment cell perimeter at different z‐levels. To understand variability in these cell area measurements due to hand segmentation, we repeated measured individual cells at one time point. We found that the variability in the data due to hand segmentation was about 5 μm^2^.

### 4.6 Statistical analysis

All statistical tests were performed with Graphpad Prism 9.5. For significance between mean values, the nonparametric Mann–Whitney post‐test was used. In all figures, statistical significance for *p*‐values (≥.05) is indicated as follows: *p <.05, **p <.01, and ***p <.001. Unless indicated, all the error bars shown in figures are SEM (standard error of the mean). Graphs in figures were generated using GraphPad Prism 9.5.

### 4.7 Tissue preparation for serial block face SEM

Embryos were embedded in agarose prior to immersion in fixative (1.5% glutaraldehyde, 2% paraformaldehyde, 10 mM sucrose, and 1 mM calcium chloride in 0.1 M sodium cacodylate buffer) at 4 degrees overnight. After several rinses with 0.1 M sodium cacodylate buffer containing 1 mM calcium chloride and 10 mM sucrose, the samples were further fixed in 2% osmium tetroxide, 1.5% potassium ferrocyanide, and 1 mM calcium chloride in 0.1 M cacodylate buffer for 1 h at 4°C. Tissue was then thoroughly rinsed in distilled water and incubated in 1% aqueous thiocarbohydrazide for 5 min. After further rinsing, the samples were treated with 2% aqueous osmium tetroxide for 30 min at room temperature, rinsed, and en‐bloc stained in 1% uranyl acetate overnight at 4°C. To enhance the contrast, the samples were incubated with Walton's lead solution for 30 min at 60°C, before proceeding to dehydration in acetone series and infiltration with Durcupan ACM resin (Sigma). After polymerization for 48 h at 60°C, tissue blocks were mounted on aluminum pins using conductive glue (CircuitWorks Conductive Epoxy) and trimmed accordingly. Before imaging, samples were gold‐coated to increase electron conductivity. The specimens were then placed inside a JEOL field emission scanning electron microscope (JSM‐7800F) equipped with a Gatan 3View system and OnPoint detector (Gatan). Section thickness was set at 50 nm (Z resolution). Samples were imaged at 2 kV under high vacuum, with an image size of 4180 × 10,174 and a final pixel size of 18 nm.

### 4.8 3D reconstructions from SBFSEM data

Raw collections of 2D SBFSEM images were aligned, adjusted, and manually segmented using the ImageJ plugin TrakEM2 software.^65^ For 3D reconstruction, raw models generated in ImageJ were exported to Blender software v.279 (www.blender.org) with Neuromorph toolset for morphometric analysis.^66^

## Supporting information


**Data S1.** Supplementary information.

**Movie S1.** Cell tracking during neural plate convergence. Dorsal view time‐lapse movie highlighting four rows of wild‐type neural plate cells tracked during convergence toward the midline (from 11 to 11.5 hpf) in a Cdh2‐GFP transgenic embryo. Colored dots indicate the centroids of tracked cells as they move toward the midline (right). Encircled centroids at the beginning of the movie indicate cells that will internalize. Arrowhead indicates neural plate midline. Anterior is top and right is medial. Frames are every 30 s. Scale bar 20 μm.

**Movie S2.** Cdh2 organization at the superficial neural plate surface. Dorsal view time lapse from a Cdh2‐GFP transgenic embryo depicting punctate Cdh2 organization at the superficial surface of neural plate cells during convergence (from 11 to 11.5 hpf). Arrowhead indicates the position of the midline. Anterior is top. Frames are every 6 s. Scale bar 20 μm.

**Movie S3.** Cdh2 fusion event during cell oscillation. Dorsal view time lapse from a Cdh2‐GFP transgenic embryo depicting a Cdh2 punctate fusion dynamics. Arrowheads indicate fusion event. Frames are every 6 s. Scale bar 5 μm.

**Movie S4.** Actin surface dynamics in neural plate cells. Dorsal view time lapse of a Tg(*actb1*:GFP‐*utr*CH) transgenic embryo depicting actin dynamics at superficial surface of the neural plate at 11.5 hpf. Stationary enveloping layer (evl) cells surround the neural plate (np) cells due to curvature of embryo surface. Frames are every 15 s. Scale bar 10 μm.

**Movie S5.** Actin‐rich medial protrusions during midline convergence. Dorsal view time lapse depicting superficial actin‐rich protrusions in two adjacent neural plate cells during convergence (11.5 hpf). The magenta channel represents *utr*CH‐RFP, and the green channel represents Cdh2‐GFP. Anterior is top while medial is right. Frames are every 7 s. Scale bar 10 μm.

**Movie S6.** Actin signal beneath the neural plate surface. The actin‐binding protein utrophin shown simultaneously at the neural plate surface (0 μm, left hand frame) and three z‐levels 1.2, 2.4, and 3.6 μm deeper from the surface, visualized using the Tg(*actb1:GFP‐utrCH*) transgenic line. Frame every 15 s. Scale bar 10 μm.

**Movie S7.** Deeper cell dynamics during neural plate convergence. Dorsal view time lapse from a Cdh2‐GFP transgenic embryo focused 8 μm below the surface of the neural plate (from 11 to 11.5 hpf). Arrowhead indicates midline. Anterior is top. Frames are every 6 s. Scale bar 20 μm.

**Movie S8.** Superficial myosin‐II dynamics duringneural plate convergence and internalization. Maximal projection dorsal view time‐lapse movie depicting myosin‐II dynamics in a Tg(*actb1:myl12.1*‐GFP) transgenic embryo. Left side of frame projection includes stationary perimeters of EVL cells as well as the superficial plate cells. Right side of frame shows neural plate cells only. Arrowhead indicates the neural plate midline. Anterior is top. Frames are every 6 s. Scale bar 10 μm.

**Movie S9.** Cell dynamics and intercellular space in wild‐type and *cdh2* ^*fr7/fr7*^ neural plate cells. Dorsal view time‐lapse movies of a wild‐type (left) and *cdh2* ^*fr7/fr7*^ (right) embryos expressing membrane‐GFP during convergence (11.5 hpf). Frames are every 6 s. Scale bar 10 μm.

**Movie S10.** Cell protrusion orientation in *cdh2* ^*fr7/fr7*^neural plate cells. Maximal projection dorsal view time lapse depicting a group of cells mosaically labeled for *utr*CH‐RFP in a *cdh2* ^*fr7/fr7*^ mutant embryo. The magenta channel represents *utr*CH‐RFP, and green channel represents membrane‐GFP. Arrowhead indicates neural plate midline. Anterior is top. Frames are every 11 s.

**Movie S11.** EVL and neural plate. Time‐lapse movie imaged to give transverse section through left hand side of neural plate and overlying EVL. Neural plate cells move medially (to right) while EVL cells (at top of tissue) remain stationary. Cell membranes in magenta, cell nuclei in cyan. Frames are every 5 min. Scale bar 5 μm.

**Movie S12.** EVL and neural plate interface. Substack from serial block‐face scanning electron microscopy data. Sections are in the transverse plane showing superficial surface of neural plate and its relation to EVL cells. Three EVL cells are pseudocolored to highlight medially directed EVL protrusions in the interface between the neural plate and EVL. Scale bar 10 μm.

**Movie S13.** Actin‐rich basal EVL protrusion dynamics. Dorsal view time lapse depicting basal EVL protrusions in a Tg(Krt18:lifeact‐RFP) transgenic embryo during neural plate convergence (11.5 hpf). These EVL cells sit immediately above neural plate. Dynamic basal lamellae project from EVL cell interfaces. Frames are every 10 s. Scale bar 10 μm.

**Movie S14.** Ctnna‐expressing basal EVL protrusions. Dorsal view time lapse depicting basal protrusions on EVL cells (large cell profiles) and superficial neural plate cell membranes (smaller cell outlines in center of frame) in a Tg(*ctnna‐citrine*)^*ct3a*^ transgenic embryo during neural plate convergence (11.5 hpf). The EVL cells sit immediately above neural plate cells but are projected into same focus in this maximum intensity projection. Frames are every 5 s. Scale bar 5 μm.

**Movie S15.** EVL and neural plate protrusions. Substack from serial block‐face scanning electron microscopy data. Sections are in the transverse plane showing superficial surface of neural plate and its relation to EVL cells. One neural plate cell is pseudocolored blue, and one EVL cell is pseudocolored yellow. Both show medially directed protrusions in the interface between the neural plate and EVL. Scale bar 10 μm.
