# Supplementary material for: An analysis of contractile and protrusive cell behaviors at the superficial surface of the zebrafish neural plate
Source: Dev Dyn. 2025 Feb 22;254(10):1115–32. doi: 10.1002/dvdy.70001 (PMC12509490; doi:10.1002/dvdy.70001)
Supplement: Supplementary file 1 — Data S1. Supplementary information. [file DVDY-254-1115-s013.docx]

**Supplementary Material**

**Fig. S1. Cell tracks during convergence towards neural plate midline.** (A ) and (B) neural plate cell tracks superimposed on background of multiple exposures of Cdh2-GFP puncta over a time period of 30 minutes (A) and 80 minutes (B). Arrowheads indicate neural plate midline. Scale bar 20 µm.


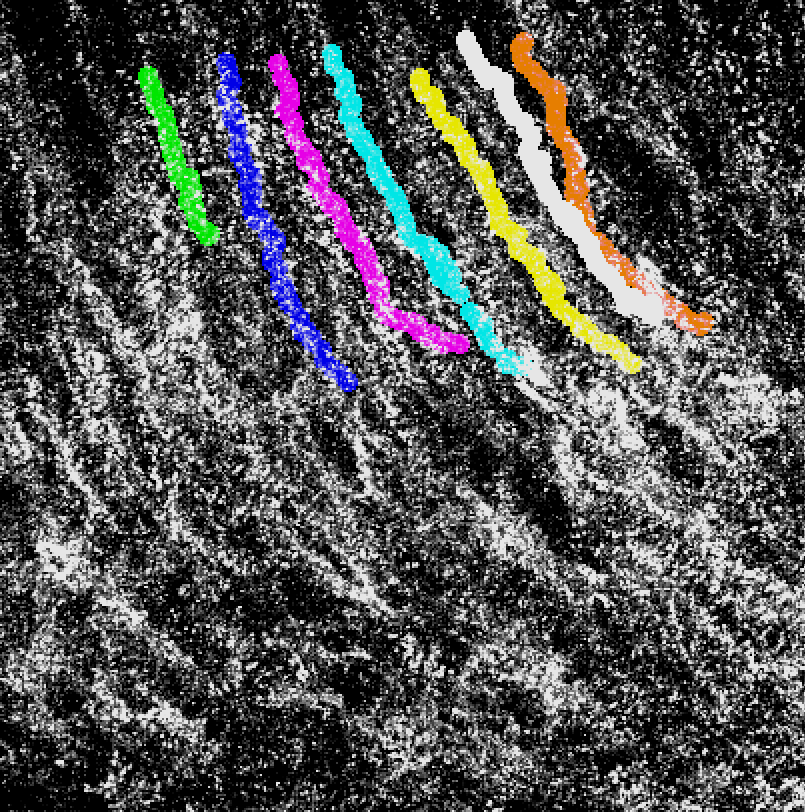

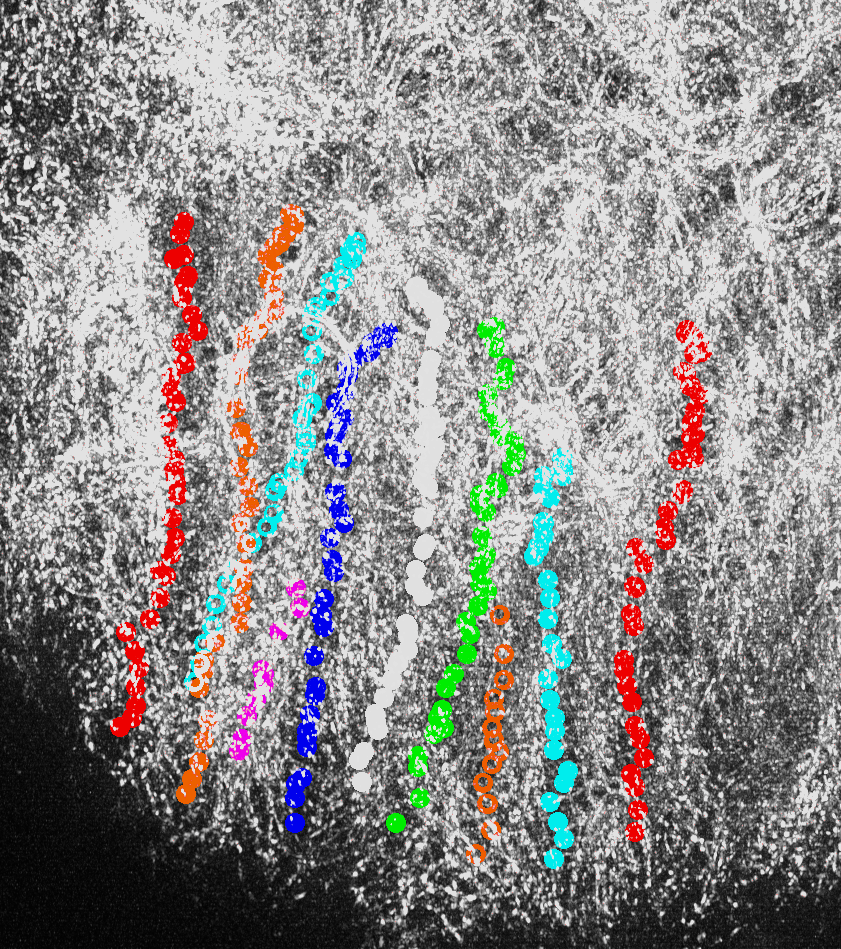


A

B

TgBAC(*cdh2*:*cdh2*-tFT) 11hpf

**Fig. S2. Cdh2 puncta fusion and distribution**

(A) Transverse view of imaging set-up. (B) Stills taken from a time-lapse of Cdh2-GFP embryos showing apparent fusion event between adjacent puncta (red arrows). Scale bar 10 µm. (C) Three z-levels from surface of neural plate and just below showing Cdh2-GFP distribution. Scale bar 5 µm.

TgBAC(*cdh2*:*cdh2*-tFT)

b


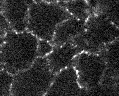

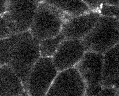


0µm

-3.5µm

C


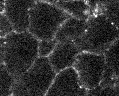

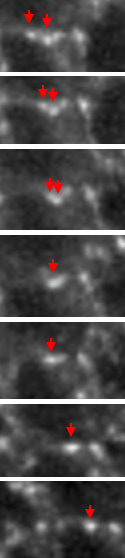


B

0 s

+46 s

+117 s

+176 s

+234 s

+436 s

+774 s

-1.5µm

A


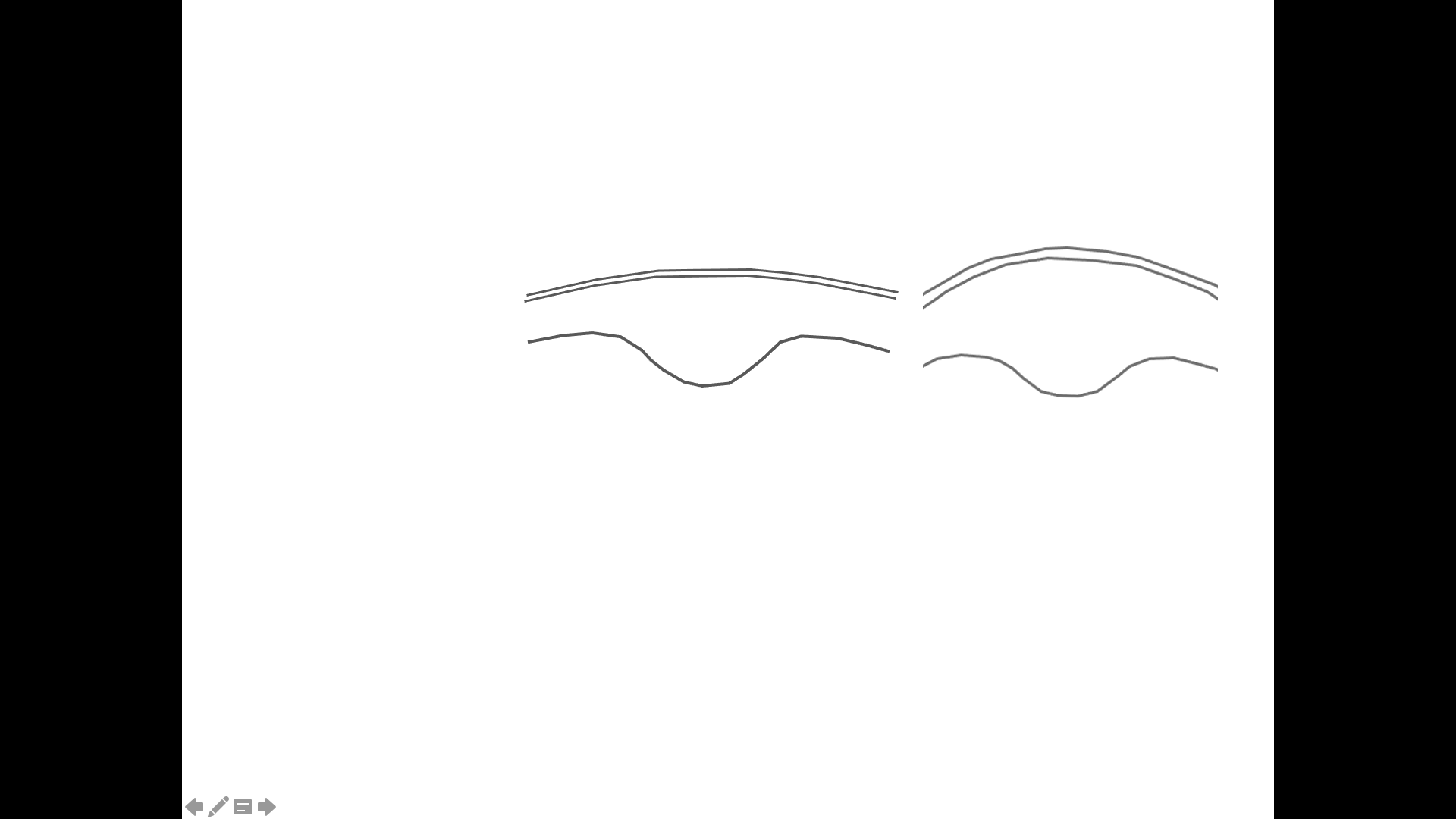


neural plate

11.5 hpf

**Fig. S3. Actin signal beneath the neural plate surface.** The actin binding protein utrophin at the neural plate surface (0 µm) and three z-levels 1.2, 2.4 and 3.6 µm deeper from the surface, visualized using the Tg(*actb1:GFP-utrCH*) transgenic line. Time-lapse activity at these levels can be seen in Movie 6. Scale bar 10 µm.


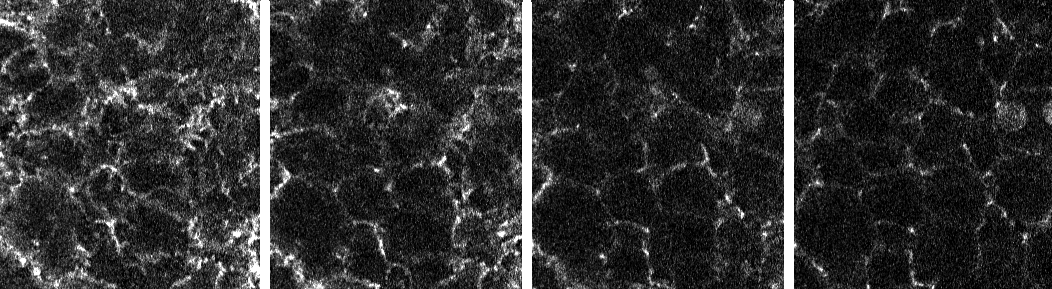


0µm

-1.2µm

-2.4µm

-3.6µm


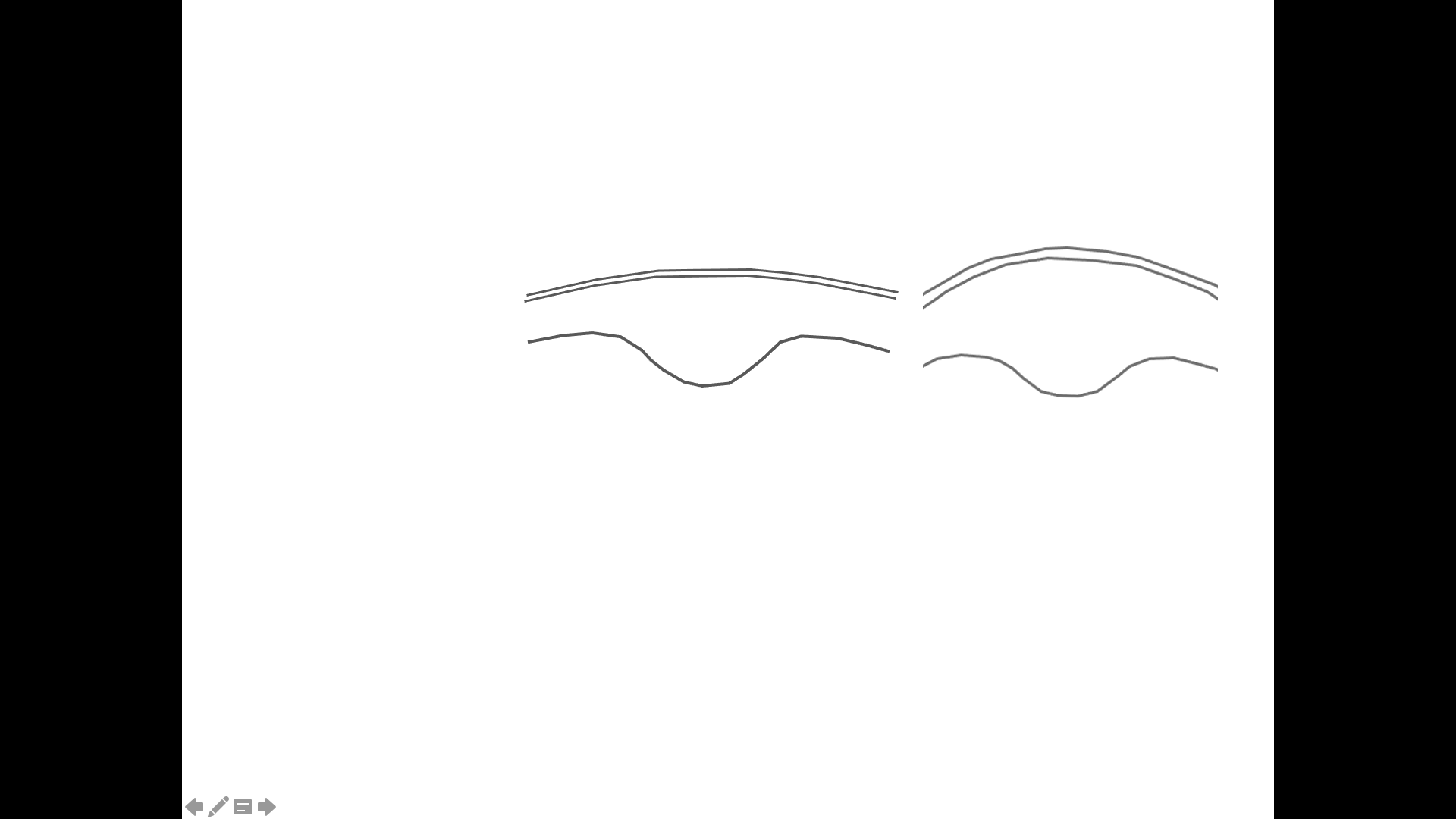


neural plate

11.5 hpf

Tg(*actb1*:GFP-*utr*CH)

**Fig. S4. Superficial versus deep oscillatory activity of the neural plate.** Box plot analysis comparing oscillation period (A), amplitude of superficial cell surface area changes during oscillations (B), and cell surface or sectional area (C) between superficial and deep levels of the neural plate. Three independent embryos were analyzed in each case. *****P*<0.0001, mean ± s.d. Colour bars indicate; o (oscillating, pale, blue), ows (oscillating while shrinking, yellow), ots (oscillating then shrinking, green), and owe (oscillating while expanding, purple). (D) Colour code depicts distinctive cell surface dynamics of neural plate progenitors. (E-F) Representative *en face* view of images taken from a timelapse recording of Cdh2-GFP transgenic embryo at superficial (E) and deeper (F) levels of the neural plate. Cells are colour coded as described in D. Scale bar 20µm.

**Fig. S5. Cell behaviours in wild-type and Cdh2 mutant neural plates.** (A) to (C) Representative images at superficial levels of the neural plate taken from time-lapse of a wild-type (A) and two Cdh2 mutant (B-C) embryos labelled with membrane-GFP (mGFP). Cells are coloured by a key cell behaviour; o (oscillating), ows, (oscillating while shrinking), ots (oscillating then shrinking), owe (oscillating while expanding), no (no oscillating), and so (shrinking only). mz indicates midline zone and scale bar is 20µm. (D) Superficial cell outlines in the neural plate midline at the start and end of a 75 minute imaging sequence in wt and *cdh2^fr7/fr7^* mutant embryos. (E) Quantification of superficial cell surface areas at the midline over 75 minute imaging sequence in wt and *cdh2^fr7/fr7^* mutant embryos.

The data illustrated in Fig. S4D and E is a new analysis of the data previously published in Figure 7i of Araya et al 2019. Our new analysis more clearly illustrates the difference in surface area shrinkage between wt and *cdh2^fr7/fr7^*.
